# Supplementary material for: Gene Regulatory Network Controlling Flower Development in Spinach (Spinacia oleracea L.)
Source: Int J Mol Sci. 2024 Jun 1;25(11):6127. doi: 10.3390/ijms25116127 (PMC11173220; doi:10.3390/ijms25116127)
Supplement: Supplementary file 1 [file ijms-25-06127-s001.zip › Supplementary Figure 3.pdf]

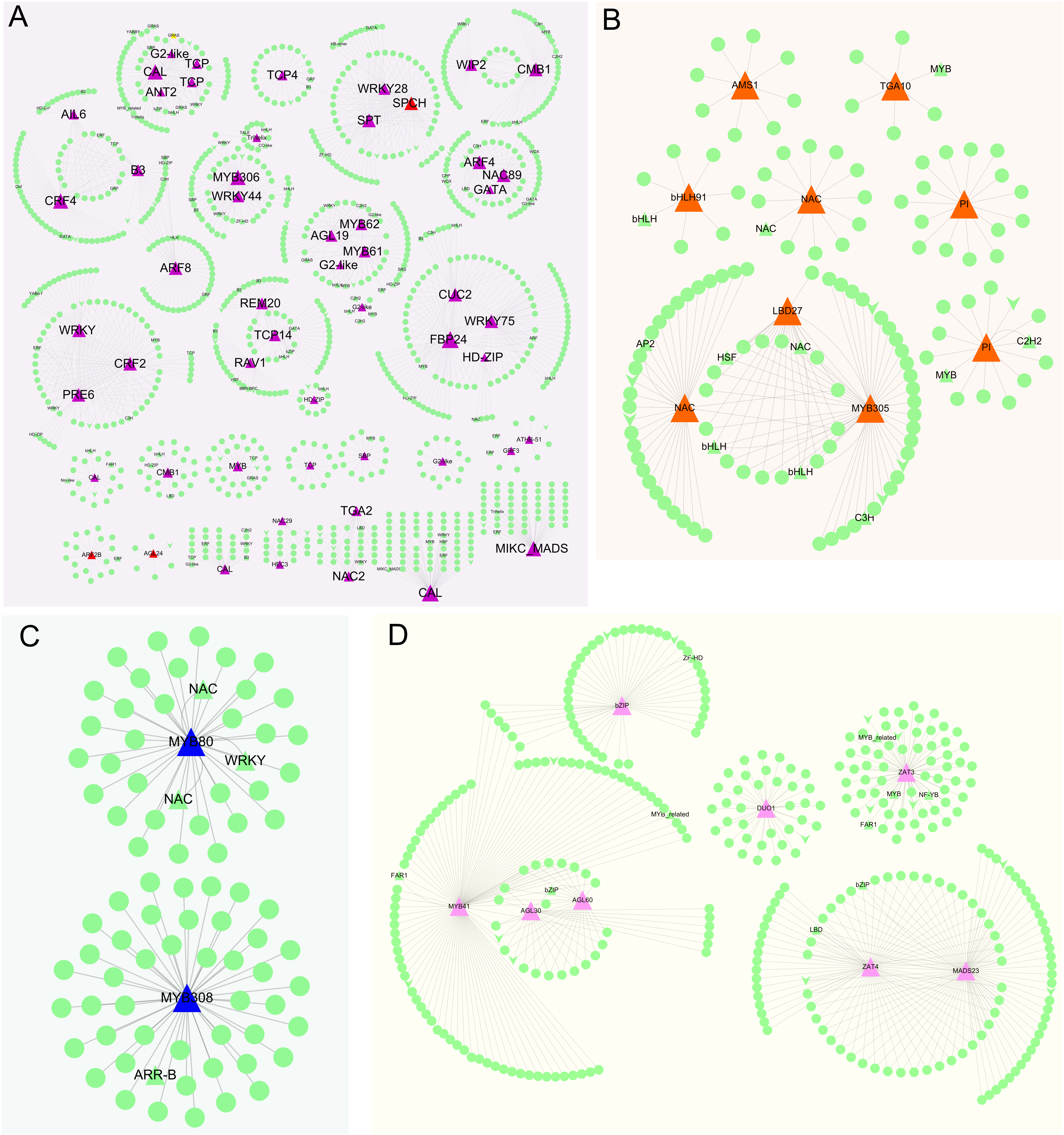

A
G2-like
TCP
CAL
TCP
ANT2
TCP4
WRKY28
SPCH
SPT
WIP2
CMB1
AIL6
B3
CRF4
MYB306
WRKY44
ARF4
NAC89
GATA
MYB62
AGL19
MYB61
G2-like
ARF8
REM20
TCP14
RAV1
CUC2
WRKY75
FBP24
HD-ZIP
WRKY
CRF2
PRE6
TGA2
NAC2
CAL
MIKC_MADS
B
AMS1
TGA10
MYB
bHLH91
NAC
PI
bHLH
NAC
LBD27
AP2
HSF
NAC
NAC
MYB305
bHLH
bHLH
C3H
PI
C2H2
MYB
C
NAC
MYB80
WRKY
NAC
MYB308
ARR-B
D
ZF-HD
bZIP
MYB_related
ZAT3
MYB
NF-YB
FAR1
DUO1
MYB_related
FAR1
MYB41
bZIP
AGL30
AGL60
bZIP
LBD
ZAT4
MADS23
