# Supplementary material for: Gene Regulatory Network Controlling Flower Development in Spinach (Spinacia oleracea L.)
Source: Int J Mol Sci. 2024 Jun 1;25(11):6127. doi: 10.3390/ijms25116127 (PMC11173220; doi:10.3390/ijms25116127)

		FS2	FS3	FS4	FS5	FS6	MS2	MS3	MS4	MS5	MS6
ABA	PP2C	59.00	46.89	35.74	6.11	6.67	46.99	20.18	5.74	0.99	0.46
	PYL	102.12	95.85	91.75	100.06	151.91	132.44	125.02	31.17	130.19	229.50
Auxin	ARF	398.35	367.23	236.92	176.04	126.88	257.09	236.23	89.22	52.73	17.63
	AUX1	115.98	182.05	209.48	158.63	97.67	210.76	202.48	47.02	21.22	8.53
	GH3	39.92	43.06	43.14	58.50	64.05	44.77	58.70	29.32	109.60	65.73
	IAA	128.94	120.49	103.07	105.46	136.06	85.48	88.02	42.77	35.01	21.51
	SAUR	19.88	12.95	10.41	18.12	36.01	11.20	10.76	35.26	167.64	440.04
BR	BRI1	48.10	45.45	61.50	53.37	45.78	34.15	29.18	37.31	18.27	12.76
	CYCD3	742.10	610.69	397.55	319.41	258.13	664.92	467.55	142.96	51.30	18.43
CK	AHP	0.00	0.00	0.00	0.00	0.00	0.00	0.00	3.13	35.31	2.00
	ARR-A	9.66	17.08	19.61	124.51	221.67	17.55	22.26	12.92	27.25	32.45
	ARR-B	1.28	1.64	2.93	2.84	2.01	1.96	2.65	18.16	11.38	9.09
	CRE1	77.59	60.48	44.35	38.17	29.74	51.89	44.52	42.96	19.12	7.83
ET	ETR	34.12	29.70	30.74	28.58	27.44	22.00	20.13	29.65	14.43	6.74
GA	DELLA	110.51	91.97	104.33	97.69	105.81	92.26	87.71	90.64	61.26	46.12
	GID1	487.36	470.34	399.08	219.50	136.02	247.23	167.62	221.09	80.54	37.76
	PIF4	2.42	2.10	2.13	3.53	4.91	1.56	2.90	11.73	4.25	2.89
JA	JAZ	369.54	421.53	337.16	505.39	847.04	369.19	304.33	121.52	137.91	140.00
	MYC2	42.48	34.70	27.15	17.37	14.88	36.23	28.52	9.59	9.80	3.13
SA	NPR1	55.68	45.12	47.55	51.83	28.69	58.28	37.07	16.17	9.76	6.35
	PR1	63.23	2.85	5.70	3.77	12.41	10.62	36.28	4.37	39.56	2226.17
	TGA	32.11	29.91	31.02	11.32	4.94	29.82	25.79	11.66	2.92	1.10

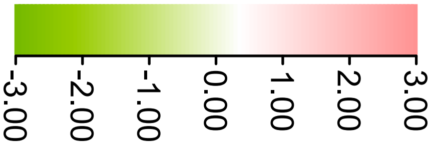

Supplement: Supplementary file 1 [file ijms-25-06127-s001.zip › Supplementary Figure 4.pdf]
