# Supplementary figures and images for: Gene Regulatory Network Controlling Flower Development in Spinach (Spinacia oleracea L.)
Source: Int J Mol Sci. 2024 Jun 1;25(11):6127. doi: 10.3390/ijms25116127 (PMC11173220; doi:10.3390/ijms25116127)

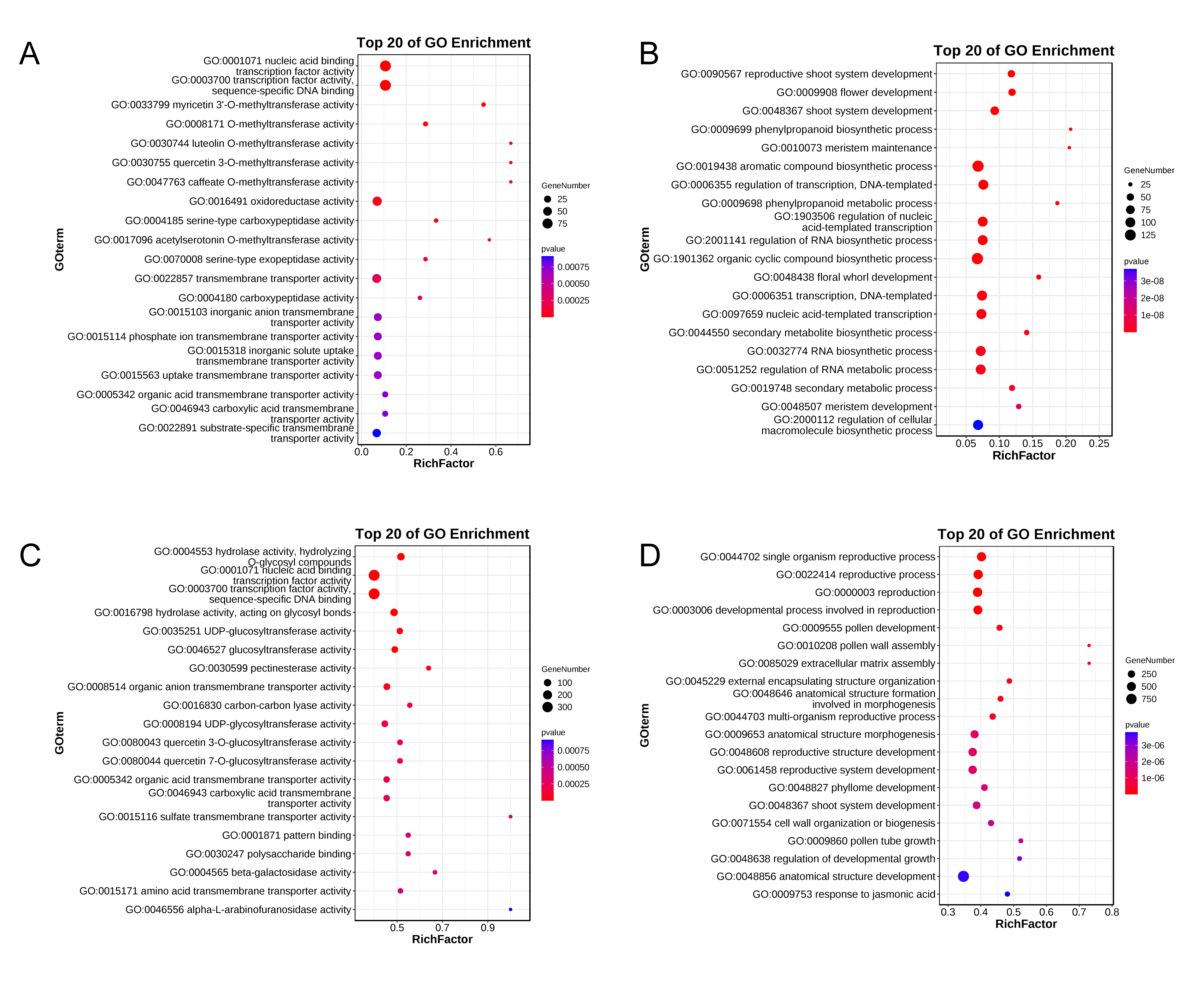

Supplement: Supplementary file 1 [file ijms-25-06127-s001.zip › Supplementary Figure 1.tif]

A

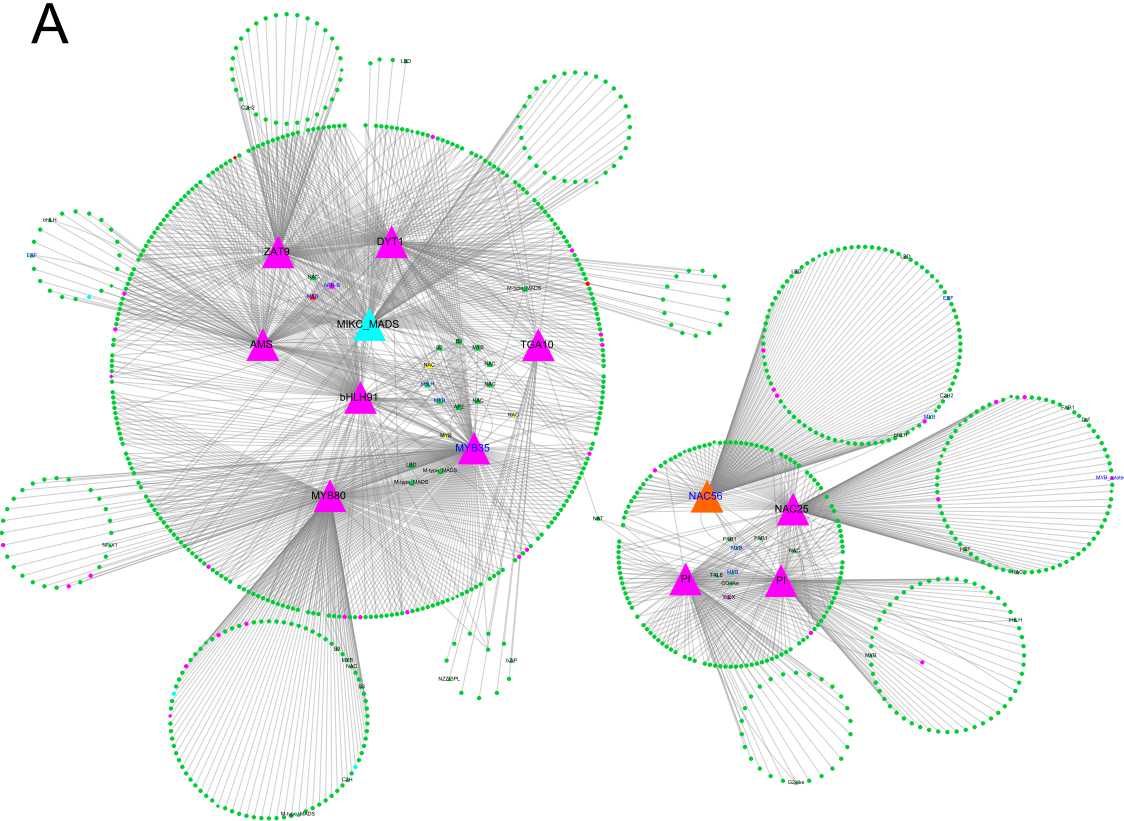

B

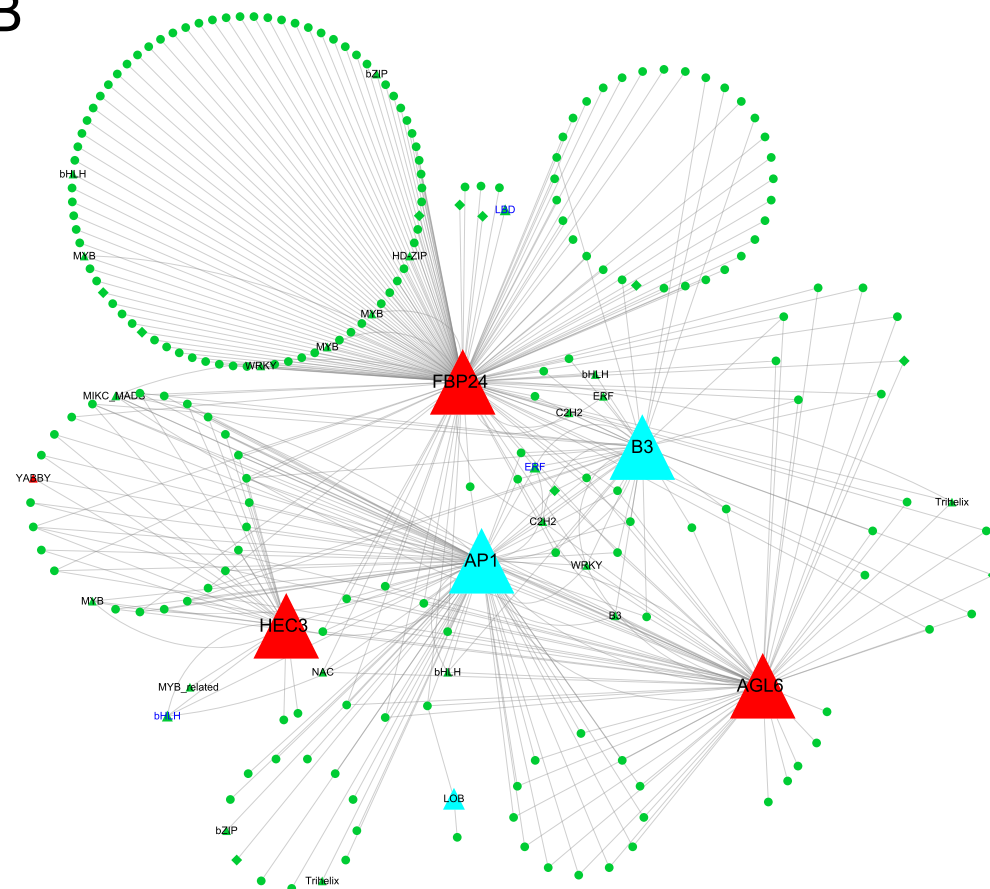

C

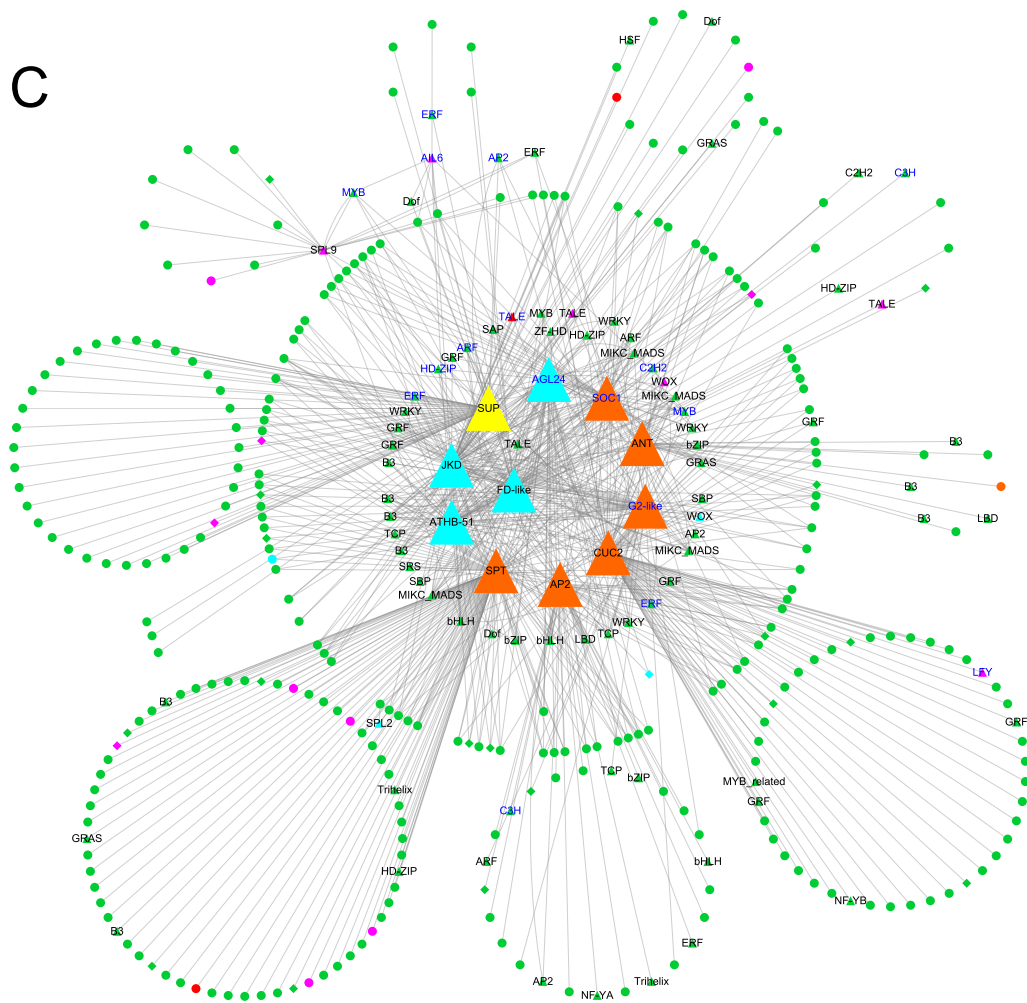

D

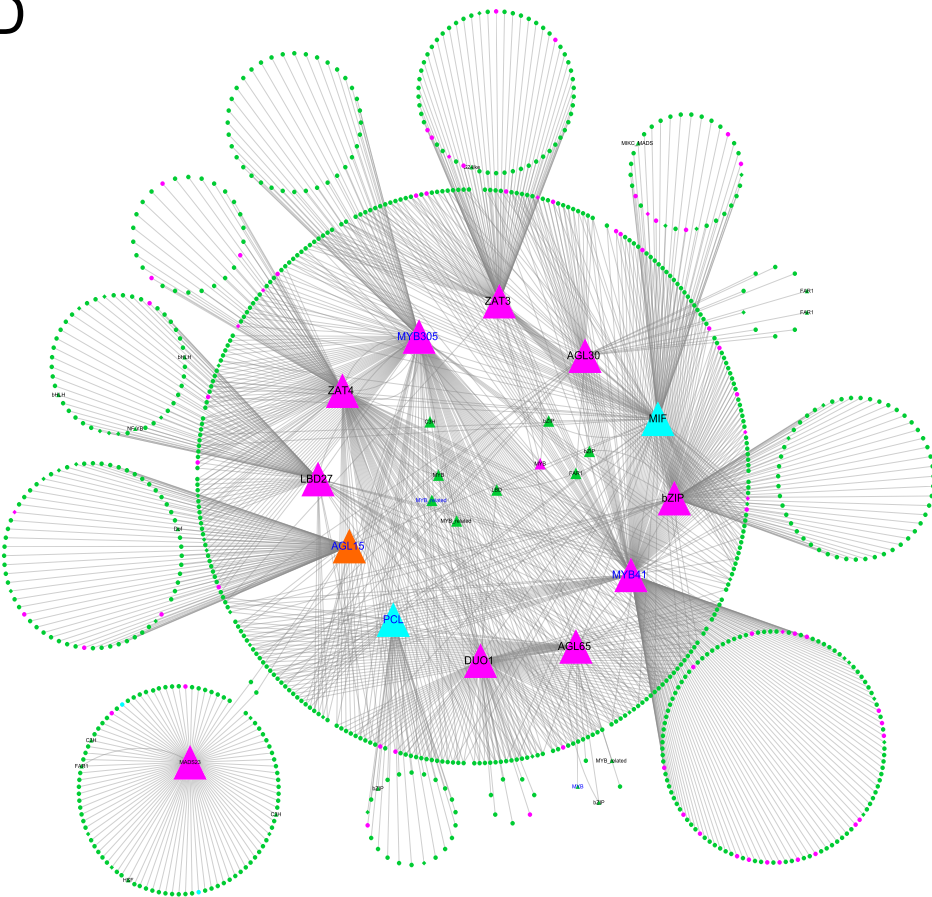

Supplement: Supplementary file 1 [file ijms-25-06127-s001.zip › Supplementary Figure 2.pdf]
